# Generation of New Knock-Out Mouse Strains of *Lysophosphatidic Acid Receptor 1*

**DOI:** 10.3390/ijms26062811

**Published:** 2025-03-20

**Authors:** Georgia Antonopoulou, Christiana Magkrioti, Ismini Chatzidaki, Dimitris Nastos, Sofia Grammenoudi, Konstantinos Bozonelos, Vassilis Aidinis

**Affiliations:** Institute for Fundamental Biomedical Research, Biomedical Sciences Research Center Alexander Fleming, 16672 Athens, Greece; georgia.antonopoulou97@gmail.com (G.A.); magkrioti@fleming.gr (C.M.); isminichatz@gmail.com (I.C.); nastos@fleming.gr (D.N.); grammenoudi@fleming.gr (S.G.); bozonelos@fleming.gr (K.B.)

**Keywords:** lysophosphatidic acid receptor 1, lysophosphatidic acid, knock-out allele, conditional deletion

## Abstract

The lysophosphatidic acid receptor 1 (LPAR1) is one of the six cognate G protein-coupled receptors of the bioactive, growth factor-like phospholipid lysophosphatidic acid (LPA). LPAR1 is widely expressed in different cell types and mediates many LPA effects. LPAR1 has been implicated in several chronic inflammatory diseases, and especially pulmonary fibrosis, where it has been established as a promising therapeutic target. Herein, we present the generation of several *Lpar1* mouse strains through genetic recombination. These strains include an initial versatile *Lpar1* strain (tm1a) from which three other strains derive: an *Lpar1* reporter knockout strain (tm1b) where LacZ has replaced exon 3 of *Lpar1*; a “floxed” *Lpar1* strain (tm1c), where exon 3 is flanked by two loxP sites allowing conditional, cell-specific *Lpar1* inactivation; and a complete KO strain of *Lpar1* (tm1d), where exon 3 has been deleted. The generated strains are novel genetic tools, that can have various applications in studying LPA-LPAR1 signaling and its role in normal physiology and disease.

## 1. Introduction

Lysophosphatidic acid (1-lyso-2-acyl- or 1-acyl-2-lyso-sn-glycero-3-phosphate, LPA) is the simplest natural glycerophospholipid. It emerges as an important bioactive lipid with growth factor-like functions that regulate key biological processes, such as proliferation, cytoskeleton reorganization, smooth muscle contraction, migration, platelet aggregation, and neurogenesis [1]. Despite its physiological actions, LPA is also involved in the pathogenesis of many inflammatory conditions and several clinical disorders including neuropathies, atherosclerosis, idiopathic pulmonary fibrosis (IPF), liver and renal fibrosis, rheumatoid arthritis (RA), and cancer [2,3,4].

LPA consists of a glycerol backbone, a free phosphate group, and a single fatty acyl chain of varying length and saturation. Thus, it is a mixture of saturated (16:0, 18:0) and unsaturated (16:1, 18:1, 18:2, 20:4) species [2]. It is produced either in the intracellular or the extracellular compartment by several enzymes. The precursor of most LPA present in biological fluids is lysophosphatidylcholine (LPC). Autotaxin (ATX) is the enzyme responsible for the extracellular hydrolysis of LPC, or of the other lysophospholipids, to LPA. LPA is water-soluble, with concentrations greater than 5 μM in the serum but lower than 1 μM in other biofluids such as plasma, saliva, cerebrospinal fluid, follicular fluid, and malignant effusions [5].

LPA binds mainly to at least six cognate receptors (LPAR1-6), to trigger its downstream signaling transduction pathways. These receptors are class A rhodopsin-like G protein-coupled receptors (GPCRs) that couple with heterotrimeric Gα subunits (G_12/13_, G_q/11_, G_i/o_, and G_s_) [1]. The *LPAR1* gene was the first among the LPARs to be identified and cloned. Its original name was ventricular zone gene-1 (*Vzg-1*) because of its enriched expression in the embryonic neuroproliferative layer of the cerebral cortex [6]. LPAR1 has been found to interact with G_12/13_, G_q/11_, and G_i/o_. Its downstream signaling cascade includes the Ras superfamily of GTPases, the serum response factor (SRF), phospholipase C (PLC), diacylglycerol (DAG), the mitogen-activated protein kinase (MAPK), and phosphatidylinositol 3-kinase-protein kinase B (PI3K). Due to the wide expression of *LPAR1/Lpar1* in many tissues and organs of both humans and mice, LPA mediates a great diversity of functions through binding to this receptor [1,7,8].

The role of LPAR1 has been primarily studied in the nervous system, where it participates in the cerebral cortex formation and function, in neuronal differentiation, proliferation of astrocytes, oligodendrocytes, and smooth muscle cells, as well as in the migration and anti-apoptosis of Schwann cells [8,9,10,11]. LPAR1 is also linked to Multiple Sclerosis, as it has been shown to shift the polarization of macrophages towards a pro-inflammatory phenotype [12].

Regarding the roles of LPAR1 outside the nervous system, it has been shown that the levels of this receptor are increased in lung inflammatory disorders, such as asthma [13] and IPF [14]. Specifically, LPA promotes fibroblast accumulation and vascular leak through LPAR1, whereas mice lacking the *Lpar1* gene were protected from modeled pulmonary fibrosis [14]. Additionally, it has been suggested that LPAR1 may promote the development of experimental dermal fibrosis through transforming growth factor beta (TGF-β) activation [15]. Moreover, LPAR1 has been identified as a contributing factor within the context of renal pathology [16], arthritis [17,18], aortic valve stenosis [19], systemic vasculitis [20], and hypertrophic cardiomyopathy [21]. Moreover, Lin et al. have highlighted the implication of LPAR1 in controlling intestinal epithelial permeability and bacterial infiltration [22]. Additionally, a single-nucleotide polymorphism in *LPAR1* has been associated with hypertension [23]. Pertaining to the role of LPAR1 in cancer, *Lpar1* mutations have been detected in liver tumors in rats [24], *LPAR1* has been suggested as a colorectal cancer risk locus [25], and the ATX-LPAR1 axis has been implicated in lung carcinogenesis [26]. LPAR1 levels have been found to be upregulated or downregulated in different types of tumors and cancer cell lines [27,28,29,30,31,32]. Finally, accumulating evidence supports the *LPAR1* participation in drug resistance, which constitutes a serious obstacle to conventional cancer therapies [32,33,34].

The aforementioned observations justify the increased attention that this receptor has gained and its proposition as a new therapeutic target. Of note, the pharmacological blockade of LPAR1 has been already revealed as a novel antifibrotic mechanism for patients with IPF [35]. In fact, the LPAR1 antagonist admilparant (BMS-986278) is currently in phase 3 clinical trials for the cure of IPF [36]. Moreover, fipaxalparant (formerly HZN-825 and SAR-100842), a small-molecule selective negative allosteric modulator of LPAR1, is in phase 2 clinical trials for systemic sclerosis [37,38]. Additionally, in mice, the LPAR1 pharmacological inhibition with a different antagonist is protective against pulmonary metastasis of osteosarcoma [39]. Furthermore, this receptor is a potential therapeutic target for RA, chronic liver disease, cardiovascular diseases, and obesity [8,18,40,41], while it has been shown that LPAR1 is a specific target of antidepressants [42,43].

The first *Lpar1-*deleted mouse strain was generated by Contos et al. [44]. The constant effort to obtain tools and mouse strains for the study of LPAR1 underlines the strong interest in this receptor in the last decades [9,45,46,47]. The available gene-targeted *Lpar1* strains have been of paramount importance in studying nervous system development and its functions, modeling human neurological diseases, and understanding bone homeostasis, renal fibrosis, as well as wound healing in colitis [22,46,48,49]. Nevertheless, it has been reported that homozygotic *Lpar1* depletion leads to 50% lethality in newborns because of craniofacial dysmorphism and suckling defects [44]. The surviving mice are smaller compared to their wild-type siblings, due to abnormal bone development, while they also present other defects, such as shortened villi in their intestine and a decreased number of proliferating epithelial cells [22]. In some studies, an *Lpar1*-null mutant that arose spontaneously during colony expansion (the “Malaga variant”) has been used. However, even though homozygotes of this variant exhibit almost complete perinatal viability, the surviving mice have altered neuronal markers, increased cortical cell death, and, in general, more pronounced defects than the original mutant, possibly due to the interaction of the *Lpar1* gene with—not fully elucidated—genetic modifiers [9,50].

Thus, the generation of versatile and well-characterized KOs for *Lpar1* that will allow the spatiotemporal depletion of *Lpar1* remains a challenging and urgent objective. Herein, we present the generation of a series of *Lpar1* KO strains, with a full KO, a reporter KO, and a conditional KO strain.

## 2. Results and Discussion

LPA is a bioactive lipid mediator that triggers several physiological events, such as cell proliferation, survival, migration, and motility, by binding mainly to specific G-protein-coupled receptors (LPAR1-6). Among the LPARs, considerable attention has been paid to LPAR1 due to its broad expression and implication in various physiological procedures, such as neurogenesis, but also in diseased states. Indeed, LPA signaling through LPAR1 has been linked to kidney, liver and lung fibrosis, metabolic and cardiovascular disorders, cancer, and drug resistance [8,51].

**Figure 1:**
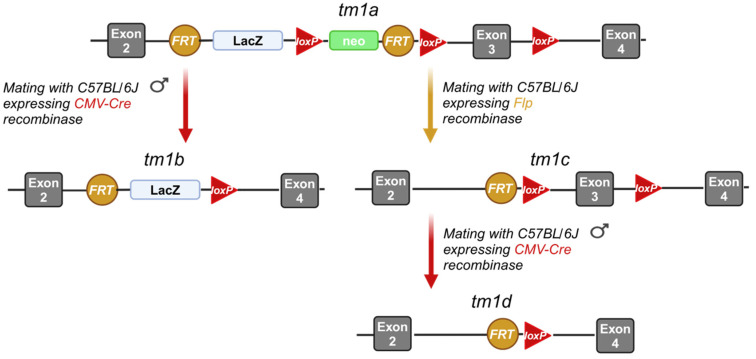
Strategy for the generation of the *Lpar1^tm1a^*, *Lpar1^tm1b^, Lpar1^tm1c^*, and *Lpar1^tm1d^* mouse strains. The third exon (called “critical exon”) of the *Lpar1* gene was loxP-flanked, while a *LacZ* reporter and a neomycin-selection cassette, including two FRT and one loxP site, were placed upstream. This allele is named “targeted mutation 1a” (tm1a). The *Lpar1^tm1b^* allele is produced by deleting the critical exon and the neomycin cassette of *Lpar1^tm1a^*, using a Cre recombinase that recognizes the loxP sites. The *Lpar1^tm1c^* allele is produced by the deletion of the *LacZ* reporter and neomycin-selection cassette of *Lpar1^tm1a^*, using a Flp recombinase that recognizes the FRT sites. The *Lpar1^tm1d^* allele is produced by deletion of the critical exon from *Lpar1^tm1c^*, using Cre recombinase; tm1a, tm1b, tm1c, and tm1d correspond to the allele nomenclature defined by EUCOMM. The recombinases (CMV-Cre or Flp) required to obtain each strain are stated next to the arrows. The color code is linked to each recombinase recognition site (loxP or FRT). The figure was prepared with Biorender under agreement number PN27QADSHN.

In this work, we present the generation of several *Lpar1* strains that could serve as valuable tools in the study of *Lpar1* implication in (patho-) physiology. The chosen approach for the generation of the desired mouse strains was based on the European Conditional Mouse Mutagenesis Program (EUCOMM)’s conditional gene targeting strategy, referred to as “targeted mutation 1a” (tm1a) [52]. As summarized in Figure 1, a *lacZ* reporter gene, a neomycin-resistance selection cassette, two FRT sites, and two loxP sites (one just before and one after the neomycin cassette) were placed upstream of the third exon (called “critical exon”) of the *Lpar1* gene locus in the murine Embryonic Stem (ES) cells obtained from EUCOMM. A third loxP site was inserted immediately after the critical exon to facilitate its removal. The two FRT sites that are inserted, one upstream of the *lacZ* cassette and one upstream of the third loxP site, allow the simultaneous removal of both the lacZ and neomycin-resistance cassettes.

**Figure 2:**
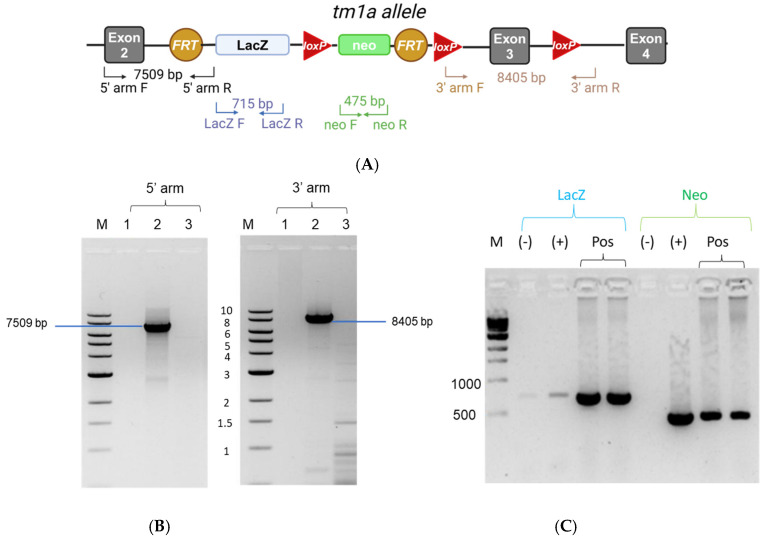
Validation of the successful *Lpar1* gene locus targeting in the ES clones derived from EUCOMM. (**A**) An illustration that indicates the positions and directions of the primers that were used to verify the *Lpar1* gene locus targeting, as well as the size of the expected products. (**B**) The products of the Long-Range PCR for the 5′ arm, at 7509 bp, and for the 3′ arm, at 8405 bp, verified the correct integration of the allele on both the 5′ and 3′ side in the ES clones derived from EUCOMM. Electrophoresis of products was performed at a 0.7% agarose gel in TAE buffer. M: 1 kb marker; 1: The PCR mixture containing H_2_O instead of DNA (Negative Control); 2: The PCR mixture containing DNA isolated from the EPD0496_2_C05 clone; 3: The PCR mixture containing DNA isolated from C57BL/6 (Wild Type) mouse. (**C**) PCR products from the amplification of the *LacZ* reporter gene and the neomycin selection cassette, at 715 bp and 475 bp, respectively, confirmed the transgenic gene locus. Electrophoresis of PCR products was performed at a 1.5% agarose gel in TBE buffer. M: marker; (−): Negative Control (the PCR mixture containing H_2_O instead of DNA); (+): The PCR mixture containing DNA isolated from the EPD0496_2_C05 clone; Pos: The PCR mixture containing DNA isolated from two different chimeras that were obtained from the pseudopregnant females in which we have transferred the blastocysts injected with EPD0496_2_C05 ES cells. Panel (**A**) was prepared with Biorender under agreement number ZT27QAE8K4.

**Figure 3:**
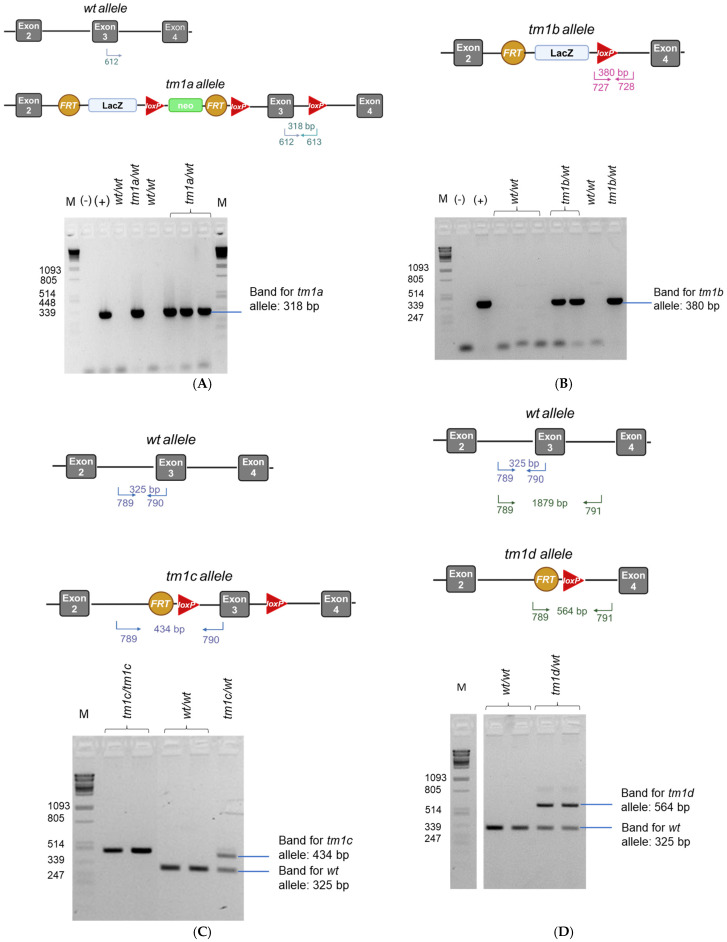
Generation of murine *Lpar1* knock-out alleles and the respective mouse strains. Genotyping strategy and representative examples of mice carrying *Lpar1* wild-type (wt, *Lpar1^wt^*) and *Lpar1^tm1a^*, *Lpar1^tm1b^, Lpar1^tm1c^*, or *Lpar1^tm1d^* alleles. Arrows indicate the positions and directions of PCR primers. (**A**) Primer 612 binds both to the *Lpar1^wt^* and *Lpar1^tm1a^* allele, while primer 613 binds only to the *Lpar1^tm1a^* allele; thus, mice bearing the *Lpar1^tm1a^* allele have a product of 318 bp, whereas the *Lpar1^wt^* allele does not produce any band. (**B**) Primers 727 and 728 bind only to the *Lpar1^tm1b^* allele and not to the *Lpar1^wt^* allele; the size of the product is 380 bp. (**C**) Primers 789 and 790 bind both to the *Lpar1^wt^* and *Lpar1^tm1c^* allele, but the sizes of the expected PCR products are different: 325 bp for the *Lpar1^wt^* allele and 434 bp for the *Lpar1^tm1c^* allele. This genotyping strategy enables the identification of *Lpar1^tm1c^* heterozygotes (*tm1c/wt*). (**D**) Primer 789 binds both to the *Lpar1^wt^* and *Lpar1^tm1d^* allele. Primer 790 recognizes a site in the critical exon; thus, it amplifies only the *Lpar1^wt^* allele and the product size is equal to 325 bp. Even though primer 791 binds to both alleles, the PCR product of 789–791 for the *Lpar1^wt^* allele is too large (1879 bp) and cannot be detected with conventional PCR. On the other hand, the PCR product of 789–791 for the *Lpar1^tm1d^* allele is 564 bp and its band is detectable. Using this genotyping strategy, the identification of *Lpar1^tm1d^* heterozygotes (*tm1d/wt*) is also possible. M: Marker, *Pstl*-cut lambda DNA. Separate panels of the figure were prepared with Biorender under agreement numbers PN27QADSHN, NZ27QAGWYB, and MA27QAFHMT.

Initially, in order to verify the correct integration of the tm1a allele in the ES clones derived from EUCOMM, we performed Long-Range PCRs to amplify the 5′ arm and the 3′ arm. This type of PCR allows the detection of large DNA fragments that cannot be amplified with conventional PCR. According to the electrophoresis (Figure 2B), the size of the Long-Range PCR products for the 5′ arm (7509 bp) and the 3′ arm (8405 bp) was as expected, validating the appropriate integration of the transgene in the *Lpar1* gene locus. For the detection of the *LacZ* reporter gene and neomycin selection cassette, conventional genomic PCR was performed. The PCR products at 715 bp for the *LacZ* reporter gene and at 475 bp for the neomycin selection cassette verified the presence of these elements in the transgenic gene locus (Figure 2C). Upon these confirmatory results, ES cells were microinjected into C57Bl/6 albino blastocysts and transferred to pseudopregnant females. Chimeric offspring were crossed with C57BL/6 female mice to generate C57BL/6-A^tm1Brd^Lpar1^tm1a(EUCOMM)Wtsi^/Flmg which was submitted to The European Mouse Mutant Archive (EMMA) (EM:09092). Representative genotyping results for this new strain are presented in Figure 3A. The primers designed for the detection of the *Lpar1^tm1a^* allele amplified a sequence of 318 bp that is present only in the transgenic allele and not in the wild-type (*Lpar1^wt^*) allele. Heterozygous *Lpar1^tm1a^* mice were viable (Table S1), whereas homozygosity for *Lpar1^tm1a^* may lead to perinatal lethality, although a larger number of births is needed to draw safe conclusions (Table S2).

The second mouse line generated in this study was the C57BL/6-A^tm1Brd^Lpar1^tm1b(EUCOMM)Wtsi^/Flmg, which has also been submitted to EMMA (EM:10041). This strain was generated from the mating of female C57BL/6-A^tm1Brd^Lpar1^tm1a(EUCOMM)Wtsi^/Flmg mice, heterozygous for the *Lpar1^tm1a^* allele, with male Tg-CMV-Cre mice (Figure 1). As the Cre recombinase is ubiquitously expressed under the control of the Cytomegalovirus (CMV) promoter [53], the recombination occurs in all cells and tissues of the offspring. For the genotyping PCR, the pair of primers was designed to amplify a sequence that is part of the transgenic *Lpar1* locus of the *Lpar1^tm1b^* allele (product size at 380 bp), but it is not present in the *Lpar1^wt^* allele (Figure 3B).

**Figure 4:**
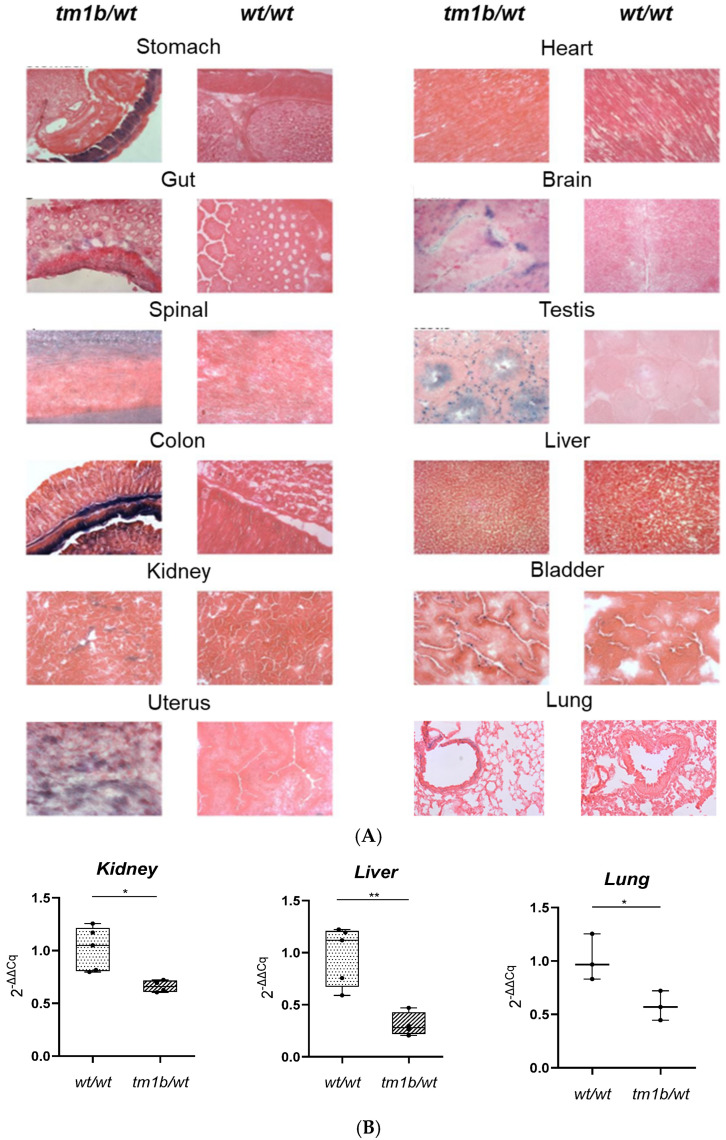
*Lpar1^tm1b/wt^* mouse strain characterization. (**A**) *Lpar1* expression varies among the different murine tissues according to the X-gal staining. Given that the *LacZ* reporter gene has been placed downstream of the *Lpar1* promoter, *LacZ* expression is spatiotemporarily controlled by the *Lpar1* promoter. Representative images from *Lpar1^tm1b/wt^* and *Lpar1^wt/wt^* mice are shown. Magnification 20×. (**B**) The transcription levels of *Lpar1* are reduced by almost 50% in the kidney, liver, and lung derived from heterozygous *Lpar1^tm1b/wt^* compared to *Lpar1^wt/wt^* mice (n = 3–5, total number of animals used = 9). Real-time PCR analysis includes the normalization of *Lpar1* mRNA expression levels with the expression levels of *B2m* in different tissues. The relative quantification of the target-gene expression was done using the Livak (2^−ΔΔCq^) method and presented as the fold change of *Lpar1* in the *Lpar1^tm1b/wt^* mice relative to *Lpar1^wt/wt^* mice, * *p* < 0.05 and ** *p* < 0.01.

C57BL/6-A^tm1Brd^Lpar1^tm1b(EUCOMM)Wtsi^/Flmg strain is considered a reporter KO, because recombination at loxP sites results in the deletion of the neomycin cassette and the critical exon, but not of the *LacZ* reporter sequence (Figure 3B). Consequently, in tissues where normally the *Lpar1* promoter is active and the *Lpar1* would be expressed, the *LacZ* sequence is transcribed producing β-galactosidase even after the excision of the critical exon of *Lpar1*. Thus, the visualization of β-galactosidase with X-gal staining is indicative of the endogenous expression pattern of *Lpar1*. As shown in Figure 4A, and in line with the respective literature [1], *Lpar1* expression varied among different tissues: brain, colon, stomach, spinal cord, testis, uterus, and white adipose tissue, presenting the highest β-galactosidase expression (blue areas) at the X-gal staining. Consistent with the deletion of *Lpar1* critical exon and validating the proper gene targeting, RT-PCR revealed that the mRNA levels of *Lpar1* in kidney, lung, and liver tissues of *Lpar1^tm1b/wt^* mice were reduced by almost 50% (Figure 4B), as expected for mice carrying one *Lpar1^tm1b^* and one *Lpar1^wt^* allele. Additionally, *Lpar1^tm1b^* in a heterozygotic state may lead to perinatal lethality, as seen in Table S3.

To generate the “floxed” *Lpar1^tm1c^* allele and the respective mouse strain for conditional deletion (C57BL/6-A^tm1Brd^Lpar1^tm1c(EUCOMM)Wtsi^/Flmg), C57BL/6-A^tm1Brd^Lpar1^tm1a(EUCOMM)Wtsi^/Flmg mice were crossed, with transgenic mice expressing the FLP1 recombinase (*FlpE*) [54]. In the presence of this recombinase, the FRT-framed tm1a region is excised, removing both the *LacZ* reporter sequence and the neomycin-resistance cassette. Thus, the gene structure is restored and the derived C57BL/6-A^tm1Brd^Lpar1^tm1c(EUCOMM)Wtsi^/Flmg strain is the “floxed” strain. The genetic recombination was verified with genotyping PCR (Figure 3C); the expected and observed size of the PCR products was 325 bp for the *Lpar1^wt^* allele and 434 bp for the *Lpar1^tm1c^* allele. *Lpar1^tm1c/wt^* heterozygotes can be identified with this genotyping strategy (Figure 3C). *Lpar1^tm1c^* heterozygosity does not seem to affect viability (Table S4). This novel strain was also deposited to the local EMMA node (EM:09947).

**Figure 5:**
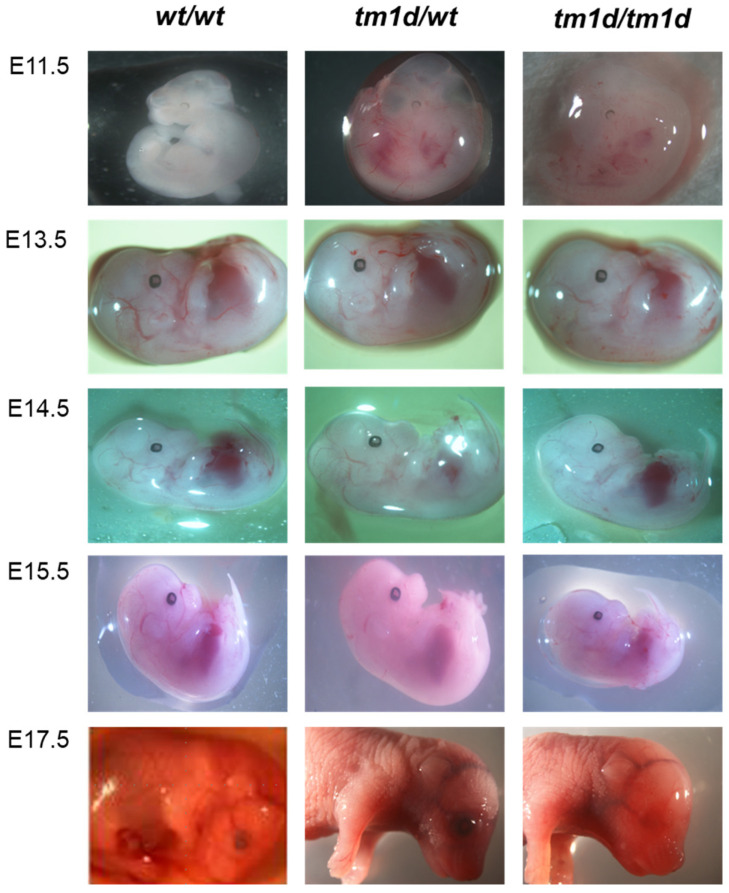
*Lpar1^wt/wt^, Lpar1^tm1d/wt^,* and *Lpar1^tm1d/tm1d^* embryos from E11.5, E13.5, E14.5, E15.5, and E17.5. No profound morphological differences can be pointed out among *Lpar1^wt/wt^*, *Lpar1^tm1d/wt^,* and *Lpar1^tm1d/tm1d^* embryos.

For the generation of the C57BL/6-A^tm1Brd^Lpar1^tm1d(EUCOMM)Wtsi^/Flmg mouse strain, we further crossed C57BL/6-A^tm1Brd^Lpar1^tm1c(EUCOMM)Wtsi^/Flmg with Tg-CMV-Cre mice, triggering the deletion of exon 3 (the critical exon) of the *Lpar1* gene (Figure 1). As above, this mouse line has been submitted to EMMA (EM:10057). With the designed primers, the length of the amplified sequence for the *Lpar1^tm1d^* allele was 564 bp, whereas the *Lpar1^wt^* allele was 325 bp (Figure 3D). Again, this genotyping strategy allows the identification of the *Lpar1^tm1d/wt^* heterozygotes. Importantly, the number of homozygous KO (*Lpar1^tm1d/tm1d^*) mice, born from the mating of heterozygous *Lpar1^tm1d/wt^* mice, was significantly smaller than the one expected from the Mendelian ratio; from the eight matings, only 6 out of the 71 offspring born were homozygotes for the *Lpar1^tm1d^* allele (Table 1 (B)) and these had smaller body size and shorter snouts. From the first *Lpar1* KO strain, it has been reported that the targeted deletion of *Lpar1* leads to neonatal lethality by approximately 50% and to impaired suckling in neonatal pups, potentially due to olfactory bulb and cerebral cortex defects [44]. The lethality linked to the homozygotic deletion of *Lpar1* has been mostly characterized as neonatal/perinatal/postnatal and not embryonic [44,45]. Indeed, as presented in Table 1 (A), the numbers of the observed E11.5, E13.5, E14.5, E15.5, and E17.5 *Lpar1^tm1d/tm1d^* embryos were almost as expected. This result, combined with the sub-Mendelian ratio of *Lpar1^tm1d/tm1d^* mice at postnatal day 7, when the tissue for the genotyping of the mice was obtained, supports the concept that the *Lpar1* depletion in homozygotic state results in pre-weaning lethality. This is also in agreement with the absence of any profound morphological abnormalities in the *Lpar1^tm1d/tm1d^* embryos (Figure 5).

**Figure 6:**
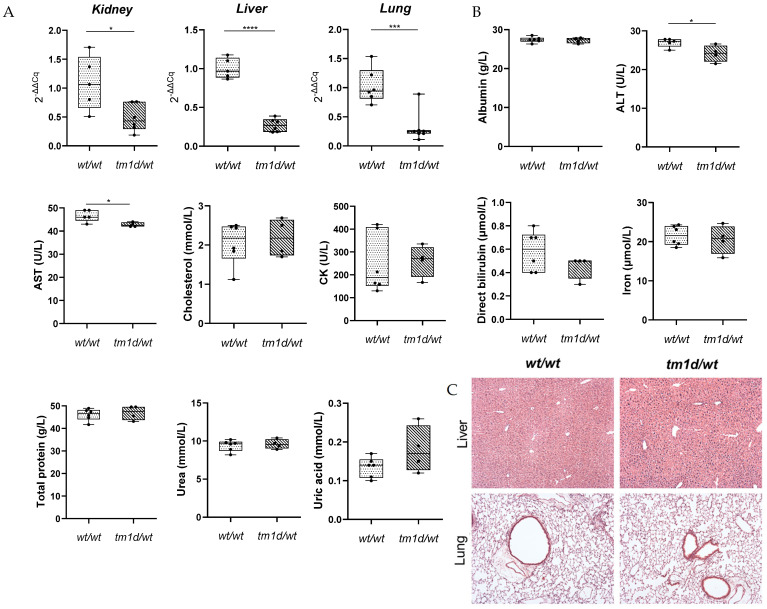
*Lpar1^tm1d/wt^* mouse strain characterization. (**A**) The transcription levels of *Lpar1* are reduced by almost 50% in the kidney, liver, and lung derived from heterozygous *Lpar1^tm1d/wt^* compared to *Lpar1^wt/wt^* mice (n = 6–7, total number of mice = 13). Real-time PCR analysis of *Lpar1* mRNA expression levels normalized to the expression levels of *B2m* in different tissues. The relative quantification of the target-gene expression was done using the Livak (2^−ΔΔCq^) method and presented as the fold change of *Lpar1* in the *Lpar1^tm1d/wt^* mice relative to *Lpar1^wt/wt^* mice. (**B**) Genetic excision of *Lpar1* in an heterozygotic state has no major effect on biochemical factors indicative of main body functions. Serum biochemical analytes from *Lpar1^tm1d/wt^* mice and *Lpar1^wt/wt^* mice were measured using a Beckman Coulter AU480 Clinical Chemistry Analyzer. ALT: Alanine transaminase; AST: Aspartate transaminase; CK: Creatine kinase (n = 4–6). (**C**) Deletion of *Lpar1* in a heterozygotic state does not affect tissue histology in adult mice, as shown by the Hematoxylin and Eosin (H&E) staining of liver and lung tissue, derived from *Lpar1^wt/wt^* and *Lpar1^tm1d/wt^* mice, fixed in formalin. Magnification 10×. * *p* < 0.05, *** *p* < 0.001 and **** *p* < 0.0001.

Similarly to the *Lpar1^tm1b/wt^* mice (Figure 4B), RT-PCR confirmed that *Lpar1* mRNA transcripts were reduced by almost 50% in *Lpar1^tm1d/wt^* mice compared to *Lpar1^wt/wt^* mice (Figure 6A). Given that the up-regulation of other LPAR genes could potentially compensate for the absence of *Lpar1*, we determined whether the transcript levels of *Lpar2-6* and other receptors of LPA (*Gpr35*, *Gpr87*, *P2y10*, *Trpv1*, *Pparγ*, and *Rage*) differentiated. We did the same for the transcripts of lipid phosphate phosphatases (*Lpp1*, *Lpp2*, *Lpp3*), the metabolic enzymes of LPA, which affect the levels of LPA and, thus, its downstream signaling. No alteration in the expression levels of these genes was observed in the kidneys of heterozygous *Lpar1^tm1d/wt^* mice (Figures S1–S3). Moreover, no significant changes in clinical biochemistry analytes indicative of liver, kidney, or pancreatic function were identified among the *Lpar1^tm1d/wt^* and *Lpar1^wt/wt^* mice, apart from a decrease in alanine transaminase and aspartate transaminase levels in the plasma of *Lpar1^tm1d/wt^* mice (Figure 6B). Additionally, as the representative H&E staining of liver and lung sections revealed, the reduction of *Lpar1* expression by approximately 50% had no effect on tissue histopathology (Figure 6C). Finally, no obvious macroscopic abnormalities were observed, and the mice were healthy and fertile without lethality. The difference between the heterozygous *Lpar1^tm1d/wt^* mice, which do not present lethality, and the heterozygous *Lpar1^tm1b/wt^* mice, which seem to present lethality, could be attributed to the different derivation of the two strains (with separate recombinases). Moreover, larger numbers of progeny would perhaps resolve this discrepancy.

The mating of e.g., *Lpar1^tm1c/tm1d^* with strains expressing a tissue-specific or inducible Cre recombinase will allow the selective and spatiotemporal *Lpar1* deletion, overcoming the restriction imposed by the pre-weaning lethality of *Lpar1^tm1d/tm1d^*. The versatility of our mouse strains has already been exploited by Ray et al. [55]. To avoid the potential developmental effects of ATX genetic deletion, this group crossed the *Enpp2^n/n^* mice (that we have generated and submitted to EMMA [56]) with the Pax7-CreER mice, ensuring that the Cre recombination will occur at the desired developmental point upon tamoxifen injection. Similarly, they have crossed the C57BL/6-A^tm1Brd^Lpar1^tm1c(EUCOMM)Wtsi^/Flmg strain, which is among the strains we present in this paper, with Pax7-CreER mice to delete *Lpar1* in Pax7 satellite cells and have confirmed the role of LPAR1 in satellite cell-mediated muscle repair [55]. Overall, the four transgenic *Lpar1* mouse strains that we present could serve as valuable tools in the study of the implication of this LPA receptor in health and disease in mice, with the expected possible translation to humans.

## 3. Materials and Methods

### 3.1. Mice Breeding

Mice were bred at the animal facilities of the BSRC “Alexander Fleming”, under specific pathogen-free conditions and housed at 20–22 °C, 55 ± 5% humidity, and a 12 h light-dark cycle. Food and water were given ad libitum. Breeding and all experimentation conformed to the Directive 2010/63/EU, as well as to the institutional and national guidelines for the care and use of laboratory animals. Mice were checked every 2 days for their overall appearance, size, conformation, coat condition, behavior, and clinical signs. Severe weight loss (greater than 20%) and the presence of increased respiratory rate, dyspnea, tremor, increased vocalization with handling, and neurological or musculoskeletal abnormalities were used as humane end points for adult mice. Abnormal skin color and absence of a milk spot were used as humane end points for pups. Littermate mice of different genotypes were housed together.

### 3.2. Generation of the Lpar1^tm1a^, Lpar1^tm1b^, Lpar1^tm1c^, and Lpar1^tm1d^ Mouse Strains

Strain *Lpar1^tm1a^* was generated by the Transgenics Facility at BSRC in the framework of the TA call of the Infrafrontier-i3 project. To maximize the chance of achieving germline transmission, two different ES clones, namely EPD0496_2_C05 and EPD0496_2_D05, mutant for the *Lpar1* allele, were obtained from EUCOMM (Lpar1^tm1a(EUCOMM)Wtsi^, MGI:4441643). In both cases, the parent cell line is JM8A3.N1 and the coat color contribution is agouti/brown. The L1L2_Bact_P cassette is inserted at Chromosome 4 (position: 58487705) upstream of *Lpar1* exon 3 (called “critical exon”). The cassette comprises an FRT site, followed by a L*acZ* reporter sequence and a loxP site, a neomycin-resistance gene under the control of the human beta-actin promoter, SV40 polyA, a second FRT site, and a second loxP site. Finally, a third loxP site is inserted downstream of the critical exon.

The ES cells received were passage 5, male, and heterozygous for the targeted mutation. Upon arrival, they were stored at −80 °C until processed. Having validated the correct targeting of the *Lpar1* locus (as described in Section 3.3), the ES cells were expanded by the Transgenics facility of the BSRC “Alexander Fleming” and karyotypic analysis verified their genetic integrity. ES cells were then microinjected into C57BL/6 albino blastocysts. These blastocysts were derived from either freshly collected embryos or frozen stocks. Following ES cell injection, blastocysts were transferred in pseudopregnant females treated with analgesics (ketoprofen 5 mg/kg) and anesthesia (inhaled isoflurane), and pups were born. Although both EPD0496_2_C05 and EPD0496_2_D05 were used for the microinjections, only the EPD0496_2_C05 clone yielded chimeric mice. The male chimeras obtained were crossed with C57BL/6 female mice to generate C57BL/6-A^tm1Brd^Lpar1^tm1a(EUCOMM)Wtsi^/Flmg (herein mostly referred to as *Lpar1^tm1a^* mouse strain for reasons of simplicity or tm1a in the figures). Offspring were genotyped for germline transmission as described below (Section 3.3). The newly generated mouse strain has been submitted to EMMA (EM:09092) and is available upon request.

The *Lpar1^tm1b^* allele is produced by the deletion of the critical exon and the neomycin cassette of the *Lpar1^tm1a^* allele, through Cre-mediated recombination. To generate C57BL/6-A^tm1Brd^Lpar1^tm1b(EUCOMM)Wtsi^/Flmg (herein mostly referred to as *Lpar1^tm1b^* mouse strain for reasons of simplicity or tm1b in the figures), female C57BL/6-A^tm1Brd^Lpar1^tm1a(EUCOMM)Wtsi^/Flmg mice, heterozygous for the *Lpar1^tm1a^* allele, were mated with male B6-Tg(CMV-cre)1Cgn mice [53]. The X-gal staining of different tissues (presented in Section 3.7) from *Lpar1^tm1b^* mice validated the expression of the *LacZ* reporter sequence. The offspring were genotyped for the gene of Cre recombinase (see Section 3.3) and further mated with C57BL/6 mice to remove the Cre recombinase from the genetic background. The generated mouse line has been deposited into the EMMA repository (EM:10041) and is available to the scientific community.

The *Lpar1^tm1c^* allele is produced by the deletion of the LacZ reporter sequence and the neomycin-selection cassette of *Lpar1^tm1a^*, using an FLP recombinase that recognizes the FRT sites. To obtain C57BL/6-A^tm1Brd^Lpar1^tm1c(EUCOMM)Wtsi^/Flmg (herein, also, referred to as *Lpar1^tm1c^* mouse strain, or “floxed” or tm1c in the figures for reasons of simplicity), *Lpar1^tm1a^* mice were mated with B6-Gt(ROSA)26Sor^tm1(FLP1)Dym^ PCR confirmed the deletion of the targeted sequence. The offspring were further mated with C57BL/6 mice to remove the FLP recombinase from their genome. This mouse strain is also available to the research community with EMMA ID: EM:09947.

Finally, the *Lpar1^tm1d^* allele occurs by the deletion of the critical exon from the *Lpar1^tm1c^* allele, through Cre-mediated recombination. For the generation of the C57BL/6-A^tm1Brd^Lpar1^tm1d(EUCOMM)Wtsi^/Flmg (herein frequently referred to as *Lpar1^tm1d^* mouse strain or tm1d in the figures for reasons of simplicity), female *Lpar1^tm1c^* mice, heterozygous for the *Lpar1^tm1c^* allele, were mated with male B6-Tg(CMV-cre)1Cgn mice. The removal of the critical exon was validated with PCR amplification. To remove the Cre recombinase from the genetic background, offspring were mated with C57BL/6 mice. Similar to all the aforementioned strains that we generated, this mouse line has been deposited to the EMMA repository (EM:10057) and is available to the scientific community upon request.

In all cases, sperm from mice heterozygous for the respective *Lpar1^tm1x^* allele has been deposited to the local node of the EMMA repository [https://www.infrafrontier.eu/] (accessed on 1 December 2024).

### 3.3. DNA Extraction, Long-Range PCR, and Genotyping PCR

In order to validate the correct homologous recombination and *Lpar1* targeting of the ES cells derived from EUCOMM, the ES cells were seeded for clonal expansion on gelatin coated-plates (G9136, Sigma-Aldrich, St. Louis, MO, USA), with Mouse Embryonic Fibroblasts as feeder layers, using KnockOut DMEM (10829018, Thermo Fisher Scientific, Waltham, MA, USA) supplemented with Leukemia Inhibitory Factor (LIF, L5158, Sigma-Aldrich, St. Louis, MO, USA). Upon expansion, cells were trypsinized (15400054, Gibco; Thermo Fisher Scientific, Waltham, MA, USA) and DNA was isolated through overnight digestion at 56 °C with 10 μg/mL Proteinase K (3115879, Roche Diagnostics, Rotkreuz, Switzerland) in lysis buffer (50 mM Tris-HCl pH 8.0, 100 mM EDTA pH 8.0, 100 mM NaCl, 1% SDS); phenol/chloroform extraction and isopropanol precipitation were performed based on standard protocols.

QIAGEN LongRange PCR Kit (206401, QIAGEN, Hilden, Germany) was used to confirm the homologous recombination between the cassette and the 5′ and 3′ sides of the targeted allele; the composition of the reaction mix and the cycling protocol for Long-Range PCR (for 0.1–10 kb products) were in accordance with the instructions of the manufacturer. Primers were designed to bind LPAR1 genomic regions upstream and downstream of the 5′ and 3′ arms of the targeting vector, respectively, and were combined with primers binding targeting cassette-specific elements such as the SV40 pA site or the *LacZ* gene; primers are listed in Table 2. The products of the Long-Range PCR were electrophorized in a 0.7% agarose gel in TAE buffer, including a 1 kb marker (N3232S, New England Biolabs, Ipswich, MA, USA). PCR targeting the LacZ reporter sequence and the neomycin cassette were performed to a final volume of 20 μL; the PCR master mix contained 1 μL (50–100 ng) DNA, 1 μL (2.5 mM) dNTPs, 1 μL (5 pmol/μL) of each primer, 1.2 μL (25 mM) MgCl_2_, 2 μL from a custom made 10× buffer [500 mM KCl, 100 mM Tris HCl (pH 9.0 at 25 °C), 1% Triton X-100], and 0.4 μL Taq Polymerase. The primers used for the *LacZ* reporter sequence and the neomycin cassette amplification are also listed in Table 2. The thermal protocol conditions to amplify the *LacZ* reporter sequence consisted of 5 min at 95 °C polymerase activation step, 30 cycles of denaturation at 95 °C for 30 s, primer annealing at 59 °C for 40 s, extension at 72 °C for 1 min, and a final extension step at 72 °C for 5 min. For the neomycin cassette, the respective cycling protocol was 94 °C for 5 min, [94 °C for 30 s, 55 °C for 40 s, 72 °C for 1 min] for 30 cycles, and 72 °C for 5 min. The PCR products were electrophorized in a 1.5% agarose gel in TBE buffer.

For the genotyping PCRs, DNA was isolated as above, either from the tissue cut through the toe-clipping of pups for identification purposes, or from the yolk sac of the isolated embryos (see Section 3.4); the composition of the genotyping PCR master mix is as described in the previous paragraph. All the pairs of primers used for these PCRs and the respective PCR conditions are presented in Table 3. The products of the genotyping PCRs were electrophorized in a 2% agarose gel, including *PstI-cut lambda DNA* (300017, GENEON GmbH, Hesse, Germany) as a marker.

### 3.4. Isolation and Imaging of E11.5–E17.5 Embryos

Embryonic day 0.5 (E0.5) is defined as noon of the day when a vaginal plug is observed. Euthanasia of pregnant mice on predetermined time-points (E11.5, E13.5, E14.5, E15.5, E17.5) was performed in a CO_2_ chamber with gradual filling. To isolate the embryos, the abdomen of the pregnant mice was cut and the uterus was removed. Τhe embryos were dissected one by one, keeping the yolk sacs intact and handled as gently as possible. Finally, embryos were transferred to a petri dish and visualized under a Nikon SMZ800 (Nikon Corp., Tokyo, Japan) stereoscope. A part of the yolk sac was used for the genotyping of the embryos, as described in the previous Section 3.3.

### 3.5. RNA Isolation-Reverse Transcription-Real Time PCR

Tissues from adult mice of both sexes were mechanically homogenized using an ULTRA-TURRAX^®^ IKA^®^ disperser (0003725000, IKA^®^-Werke GmbH, Staufen, Germany). RNA was extracted with TRI Reagent (TR118, MRC, Cincinnati, OH, USA) and treated with DNAse (RQ1 RNAse-free DNAse, Promega, Madison, WI, USA), according to the manufacturer’s instructions. The RNA concentration and purity were determined with NanoDrop^®^ ND-1000 UV-Vis Spectrophotometer (Thermo Fisher Scientific, Waltham, MA, USA), evaluating the optical density ratio at wavelengths of 260/280 nm and 260/230 nm. Samples were placed at −80 °C until further use.

First-strand cDNA was generated with the M-MLV reverse transcriptase (28025-013, Invitrogen, Thermo Fisher Scientific, Waltham, MA, USA) according to the reagent’s protocol, using 2 μg of RNA. Real-time PCR was performed on a BioRad CFX96 Touch™ Real-Time PCR Detection System (Bio-Rad, Hercules, CA, USA), with 25 ng of each cDNA, using SYBR Select Master Mix (4472913, Thermo Fisher Scientific, Waltham, MA, USA). The pairs of the primers and their annealing temperatures are listed in Table 4. Ct values were normalized against the reference gene beta-2 microglobulin (*B2m*). The relative quantification of the target-gene expression was done with the Livak (2^−ΔΔCq^) method and is presented as the fold change of each normalized target gene in the transgenic mice relative to the control mice.

### 3.6. Tissue Processing, H&E Staining, and Imaging

Fresh mouse tissues (liver, lung) from adult mice (both sexes) were fixed in 10% neutral-buffered formalin overnight at 4 °C. Tissue was processed in a histokinette and embedded in paraffin blocks. 5 μm sections were stained with Hematoxylin and Eosin (H&E) according to standard procedures and imaged with a Nikon Eclipse E800 microscope (Nikon Corp., Tokyo, Japan), attached to a Q Imaging EXI Aqua digital camera, using the Q-Capture Pro 7 software v7.0 (QImaging, Surrey, BC, Canada).

### 3.7. X-Gal Staining

#### 3.7.1. Protocol for Cryosections

Freshly isolated mouse tissues (brain, bladder, colon, gut, heart, kidney, liver, spinal cord, stomach, uterus) from adult mice were embedded in OCT and frozen in dry ice. Sections measuring 6–10 μm were prepared on a cryotome and fixed in 2% formaldehyde/0.2% glutaraldehyde for 15 min at 4 °C. Next, they were washed twice in cold PBS/2 mM MgCl_2_ for 10 min and stained overnight with X-gal staining solution (2 mg/mL X-gal in 0.1 M Na phosphate buffer pH 7.3, 0.01% Na deoxycholate, 5 mM K_3_Fe(CN)_6_, 5.7 mM K_4_Fe(CN)_6_, 2 mM MgCl_2_, 0.02% NP-40) at 37 °C in the dark. The sections were then rinsed twice in PBS/2 mM MgCl_2_ and dH_2_O for 5 min at room temperature, counterstained with eosin and, finally, visualized under the microscope.

#### 3.7.2. Protocol for Paraffin Sections (Lungs/Testis)

The lungs from adult mice were inflated with fixation buffer (0.74% formaldehyde/0.04% glutaraldehyde/0.02% NP-40) and after their isolation, they were placed in the same buffer for 2 h at 4 °C. Testis was also placed in the fixation buffer. Both tissues were then rinsed with PBS and dH_2_O and incubated with X-gal staining solution (1 mg/mL X-gal in 0.01% Na deoxycholate, 5 mM K_3_Fe(CN)_6_, 5.7 mM K_4_Fe(CN)_6_, 4.2 mM MgCl_2_, 0.1% NP-40) for 24 h at room temperature. Tissues were rinsed twice with dH_2_O and then placed in post-fixative solution pH = 7.2 (0.1 M NaH_2_PO_4_, 0.07 M NaOH, 4% formaldehyde/1% glutaraldehyde) for several hours. After that, they were washed with PBS and embedded in paraffin. The resulting 5-µm sections were counterstained with eosin and visualized under the microscope; tissue imaging was performed as above (Section 3.6).

### 3.8. Biochemical Analysis

After the euthanasia of adult mice, blood was collected from the inferior vena cava and left for 30 min at room temperature to clot. Samples were centrifuged for 10 min at 1000× *g*/8 °C. The supernatant was transferred into a new 1.5 mL Eppendorf tube and it was centrifuged at 3500× *g*/8 °C. After repeating the previous step, serum was transferred into a new 1.5 mL Eppendorf tube and stored at −20 °C. Biochemical analysis was performed using ½ diluted serum, with a Beckman Coulter AU480 Clinical Chemistry Analyzer (Beckman Coulter, Brea, CA, USA), based on the BSRC “Alexander Fleming” phenotyping facility for the estimation of Alanine Transaminase (ALT, OSR6007, Beckman Coulter, Brea, CA, USA), Aspartate Aminotransferase (AST, OSR6009, Beckman Coulter, Brea, CA, USA), Albumin (OSR6102, Beckman Coulter, Brea, CA, USA), Cholesterol (OSR6116, Beckman Coulter, Brea, CA, USA), Creatine kinase (CK, OSR6179, Beckman Coulter, Brea, CA, USA), Direct bilirubin (OSR6111, Beckman Coulter, Brea, CA, USA), Iron (OSR6186, Beckman Coulter, Brea, CA, USA), Total protein (OSR6132, Beckman Coulter, Brea, CA, USA), Triglycerides (OSR60118, Beckman Coulter, Brea, CA, USA), Urea (OSR6134, Beckman Coulter, Brea, CA, USA), and Uric acid (OSR6098, Beckman Coulter, Brea, CA, USA) levels.

### 3.9. Statistical Analysis

Sample sizes were calculated with Power analysis using R language with mRNA levels being the primary outcome measure. The statistical analysis and graph preparation was done with GraphPad 8.0.1 (GraphPad Software Inc., Insight Partners, New York City, NY, USA). Statistical significance was assessed with the unpaired *t*-test or Welch’s *t*-test, depending on the equality of standard deviation between the different groups and with Mann–Whitney in the case of non-normal distribution (based on the Shapiro-Wilk test). Animals or data values at specific assays were excluded only when they were indicated as outliers by GraphPad.

### 3.10. Image Creation

Illustrations of Figure 1, Figure 2 and Figure 3 were created with BioRender.com, with agreement numbers PN27QADSHN, NZ27QAGWYB, MA27QAFHMT, and ZT27QAE8K4.

## Figures and Tables

**Table 1:** The *Lpar1* depletion leads to neonatal/postnatal lethality in a homozygotic state. Numbers of expected and observed (**A**) E11.5, E13.5, E14.5, E15.5, E17.5 embryos and (**B**) Seven-day-old pups, obtained by crossing heterozygous *Lpar1^tm1d/wt^* mice. Genotyping PCRs were performed using part of the yolk sac of the isolated embryos or, in the case of pups, the tissue cut through the toe-clipping for identification purposes.

(**A**)							
**Total Matings:** **6**		**Total Progeny**					
		**49 (100%)**					
		***wt/wt***		***tm1d/wt***		***tm1d/tm1d***	
	**Embryonic** **Day**	**Embryos**	**Expected**	**Embryos**	**Expected**	**Embryos**	**Expected**
		15	12–13	24	24–25	10	12–13
		30.6%	25%	48.9%	50%	20.4%	25%
E11.5		8					
		3	2	3	4	2	2
		37.5%	25%	37.5%	50%	25%	25%
E13.5		8					
		1	2	6	4	1	2
		12.5%	25%	75%	50%	12.5%	25%
E14.5		11					
		6	2–3	4	5–6	1	2–3
		54.5%	25%	36.4%	50%	9.1%	25%
E15.5		12					
		4	3	4	6	4	3
		33.3%	25%	33.3%	50%	33.3%	25%
E17.5		4					
		0	1	2	2	2	1
		0%	25%	50%	50%	50%	25%
E17.5		6					
		1	1–2	5	3	0	1–2
		16.7%	25%	83.3%	50%	0%	25%
(**B**)							
**Total Matings**		**Total Progeny**					
**8**		**71 (100%)**					
		***wt/wt***		***tm1d/wt***		***tm1d/tm1d***	
		**Born**	**Expected**	**Born**	**Expected**	**Born**	**Expected**
		20	17–18	44	35–36	6	17–18
		28.1%	25%	61.9%	50%	8.5%	25%
***1*** *♂* ***tm1d/wt × 2*** *♀* ***tm1d/wt***		**15**					
		7	3–4	7	7–8	1	3–4
		46.7%	25%	46.7%	50%	6.7%	25%
***1*** *♂* ***tm1d/wt × 2*** *♀* ***tm1d/wt***		**8**					
		1	2	6	4	1	2
		12.5%	25%	75%	50%	12.5%	25%
***1*** *♂* ***tm1d/wt × 2*** *♀* ***tm1d/wt***		**6**					
		0	1–2	6	3	0	1–2
		0%	25%	100%	50%	0%	25%
***1*** *♂* ***tm1d/wt × 2*** *♀* ***tm1d/wt***		**6**					
		4	1–2	2	3	0	1–2
		66.7%	25%	33.3%	50%	0%	25%
***1*** *♂* ***tm1d/wt × 2*** *♀* ***tm1d/wt***		**4**					
		2	1	2	2	0	1
		50%	25%	50%	50%	0%	25%
***1*** *♂* ***tm1d/wt × 2*** *♀* ***tm1d/wt***		**6**					
		1	1–2	4	3	1	1–2
		16.7%	25%	66.7%	50%	16.7%	25%
***1*** *♂* ***tm1d/wt × 2*** *♀* ***tm1d/wt***		**14**					
		3	3–4	8	7	2	3–4
		21.4%	25%	57.1%	50%	14.3%	25%
***1*** *♂* ***tm1d/wt × 2*** *♀* ***tm1d/wt***		**10**					
		2	2–3	7	5	1	2–3
		20%	25%	70%	50%	10%	25%

**Table 2:** List of primers used for the validation of the *Lpar1* targeting of the ES cells derived from EUCOMM.

Reaction and Expected Product Size	Primer	Sequence (5′→3′)
Long-Range PCR for 5′ arm (7509 bp)	5′ arm F	TCATTCCTCCTCTTAGGCTCAACC
	5′ arm R	TGGGATAGGTCACGTTGGTGTAGA
Long-Range PCR for 3′ arm (8405 bp)	3′ arm F	CCCATGGATAGCCTATCCACTCCCTCATGC
	3′ arm R	CACACCTCCCCCTGAACCTGAAAC
PCR for *LacZ* reporter gene (715 bp)	LacZ F	GATCCCGTCGTTTTACAACGTCGT
	LacZ R	GAACTTCAGCCTCCAGTACAGCGC
PCR for neomycin cassette (475 bp)	neo F	ATTGAACAAGATGGATTGCAC
	neo R	CGTCCAGATCATCCTGATC

**Table 3:** List of primers, along with the respective PCR conditions, used for the genotyping and the validation of *Cre* and *FlpE* recombinases removal from the different mouse strains generated.

Genotyping and Expected Product(s) Size	Primer	Sequence (5′→3′)	Conditions
*Lpar1^tm1a^* wt allele: No band tm1a allele: 318 bp	612	GAGGATGTCTCGGCATAGTTCTGG	$\begin{array}{l} \;95\;{}^{\circ}C\;\mathrm{for}\;3\;\min\\ \begin{array}{l} 95\;{}^{\circ}C\;\mathrm{for}\;30\;s\\ 62\;{}^{\circ}C\;\mathrm{for}\;30\;s\\ 72\;{}^{\circ}C\;\mathrm{for}\;1\;\min \end{array}\}35\mathrm{cycles}\\ \;72\;{}^{\circ}C\;\mathrm{for}\;3\;\min \end{array}$
	613	TGAACTGATGGCGAGCTCAGACC	
*Lpar1^tm1b^* wt allele: No band tm1b allele: 380 bp	727	CGGTCGCTACCATTACCAGT	$\begin{array}{l} \;94\;{}^{\circ}C\;\mathrm{for}\;5\;\min\\ \begin{array}{l} 94\;{}^{\circ}C\;\mathrm{for}\;30\;s\\ 58\;{}^{\circ}C\;\mathrm{for}\;30\;s\\ 72\;{}^{\circ}C\;\mathrm{for}\;45\;s\; \end{array}\}35\mathrm{cycles}\\ \;72\;{}^{\circ}C\;\mathrm{for}\;5\;\min \end{array}$
	728	ACTGATGGCGAGCTCAGACC	
*Lpar1^tm1c^ & Lpar1^tm1d^* *Lpar1^tm1c^:* 789, 790 wt allele: 325 bp and 1879 bp, tm1c allele: 434 bp *Lpar1^tm1d^:* 789, 790, 791 wt allele: 325 bp, tm1d allele: 564 bp	789	GGATGCTATTTCTGGGGATGA	$\begin{array}{l} \;95\;{}^{\circ}C\;\mathrm{for}\;5\;\min\\ \begin{array}{l} 95\;{}^{\circ}C\;\mathrm{for}\;30\;s\\ 60.7\;{}^{\circ}C\;\mathrm{for}\;30\;s\\ 72\;{}^{\circ}C\;\mathrm{for}\;45\;s\; \end{array}\}35\mathrm{cycles}\\ \;72\;{}^{\circ}C\;\mathrm{for}\;5\;\min \end{array}$
	790	ATACCCAATGCAGCCAAAAA	
	791	TCATGGACACTTGGACTAATGAA	
*Cre* gene (233 bp)	Cre-F	CATTTGGGCCAGCTAAACAT	$\begin{array}{l} \;94\;{}^{\circ}C\;\mathrm{for}\;5\;\min\\ \begin{array}{l} 94\;{}^{\circ}C\;\mathrm{for}\;30\;s\\ 58\;{}^{\circ}C\;\mathrm{for}\;30\;s\\ 72\;{}^{\circ}C\;\mathrm{for}\;45\;s\; \end{array}\}35\mathrm{cycles}\\ \;72\;{}^{\circ}C\;\mathrm{for}\;5\;\min \end{array}$
	Cre-R	TAAGCAATCCCCAGAAATGC	
*FlpE* gene (600 bp)	FlpE-F	AGGGGCATACAGTACCAGAT	
	FlpE-R	CCACACAGGGTTCCTTGTTT	

**Table 4:** Primers for RT-qPCR. The thermal-cycling conditions for the 40-cycle amplification were at 95 °C for 10 s and at the mentioned annealing temperature for 45 s.

Target Gene	Forward Primer (5′→3′)	Reverse Primer (5′→3′)	Annealing Temperature (°C)
*B2m*	TTCTGGTGCTTGTCTCACTGA	CAGTATGTTCGGCTTCCCATTC	60
*Lpar1*	GAGGAATCGGGACACCATGAT	TGAAGGTGGCGCTCATCT	59
*Lpar2*	GACCACACTCAGCCTAGTCAAGAC	CAGCATCTCGGCAGGAAT	58
*Lpar3*	GCTCCCATGAAGCTAATGAAGACA	TACGAGTAGATGATGGGG	59
*Lpar4*	AGTGCCTCCCTGTTTGTCTTC	GCCAGTGGCGATTAAAGTTGTAA	53
*Lpar5*	ACCCTGGAGGTGAAAGTC	GACCACCATATGCAAACG	54
*Lpar6*	GATCACTCTCTGCATCGCTGTTTC	CCCTGAACTTCAGAGAACCTGGAG	65
*Gpr35*	AAGGCCCACCTGGAGTAGAA	CCACGTGAGGGTGCTGTTAC	59
*Gpr87*	GGCCGCCACAATGAAAGAAAT	AAGAAACGCTTGGGGAGAGG	59
*Trpv1*	GGCCGAGTTTCAGGGAGAAA	TATCTCGAGTGCTTGCGTCC	59
*P2y10*	GGATGCAGTGGTTCTGGTCA	AGCAATTGGTGGGTGTTTCA	59
*Rage*	GGTCCACTGGATAAAGGATGGTG	TTTCCTGAGGTCCGTGGCTA	59
*Pparg*	GCTCGCAGATCAGCAGACTCT	GAGAAGCTGTTGGCGGAGAT	59

## Data Availability

The original contributions presented in this study are included in the article/Supplementary Materials. Further inquiries can be directed to the corresponding author.

## Supplementary Materials

The supporting information can be downloaded at: https://www.mdpi.com/article/10.3390/ijms26062811/s1.

## Abbreviations

The following abbreviations are used in this manuscript.

ATX	autotaxin
B2m	beta-2 microglobulin
CMV	Cytomegalovirus
DAG	Diacylglycerol
EMMA	European Mouse Mutant Archive
ES cells	Embryonic stem cells
EUCOMM	European Conditional Mouse Mutagenesis Program
IPF	Idiopathic pulmonary fibrosis
KO	knockout
LPA	Lysophosphatidic acid
LPAR1	Lysophosphatidic acid Receptor 1
MAPK	mitogen-activated protein kinase
PI3K	phosphatidylinositol 3-kinase-protein kinase B
PLC	phospholipase C
RA	rheumatoid arthritis
SRF	serum response factor
tm1a	targeted mutation 1a
TGF-β	transforming growth factor beta
