# Supplementary material for: Generation of New Knock-Out Mouse Strains of Lysophosphatidic Acid Receptor 1
Source: Int J Mol Sci. 2025 Mar 20;26(6):2811. doi: 10.3390/ijms26062811 (PMC11942715; doi:10.3390/ijms26062811)
Supplement: Supplementary file 1 [file ijms-26-02811-s001.zip › ijms-3428319-supplementary.pdf]

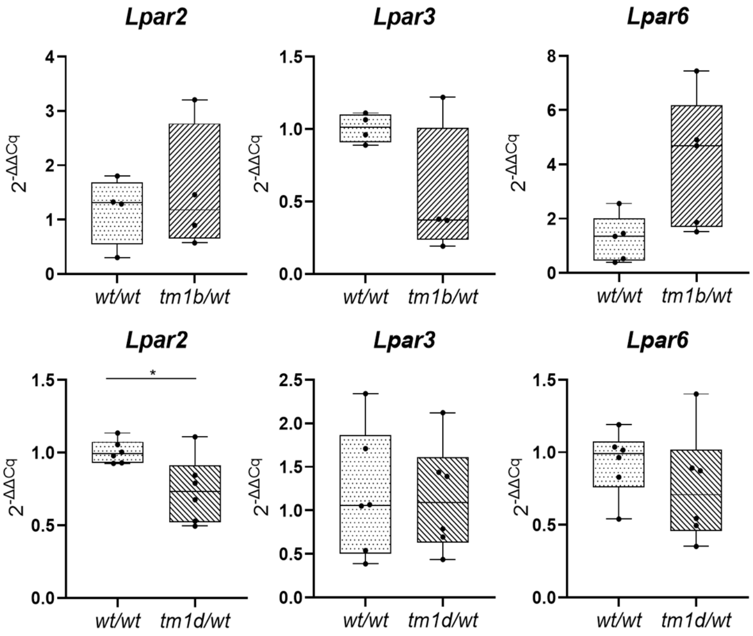

Figure S1: *Lpar*-receptor profiling in kidneys of *Lpar1*$^{tm1b/WT}$ and *Lpar1*$^{tm1d/WT}$ mice. Normalization was performed against *B2m*. Expression of *Lpar4* and *Lpar5* was not detected.

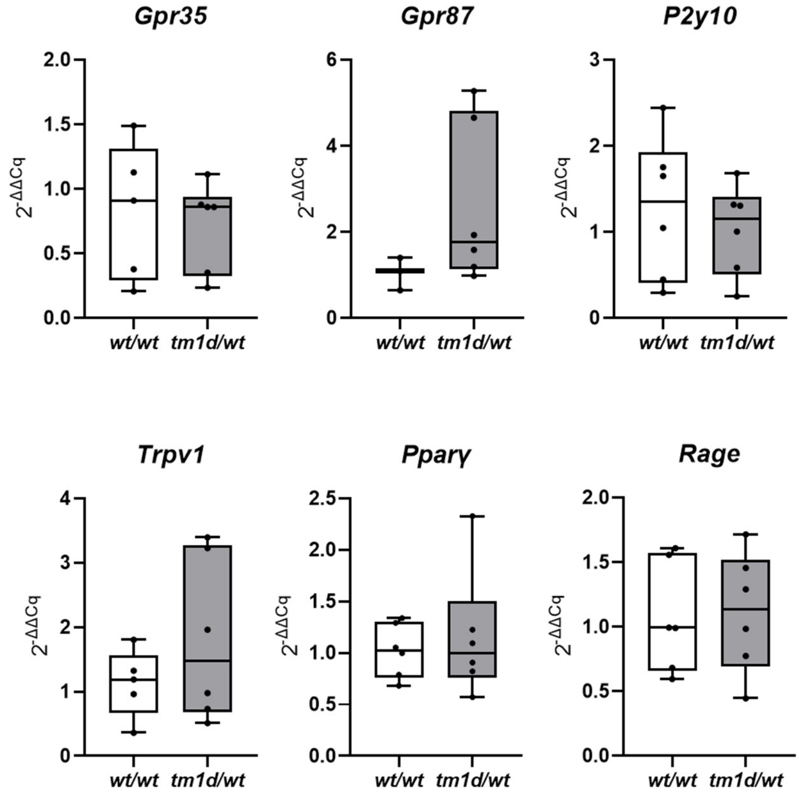

Figure S2: Profiling of *Gpr35, Gpr87, P2y10, Trpv1, Pparγ* and *Rage* as LPA receptors (other than *Lpars*) in kidneys of *Lpar1*$^{tm1d/WT}$ and control mice. Normalization was performed against *B2m*.

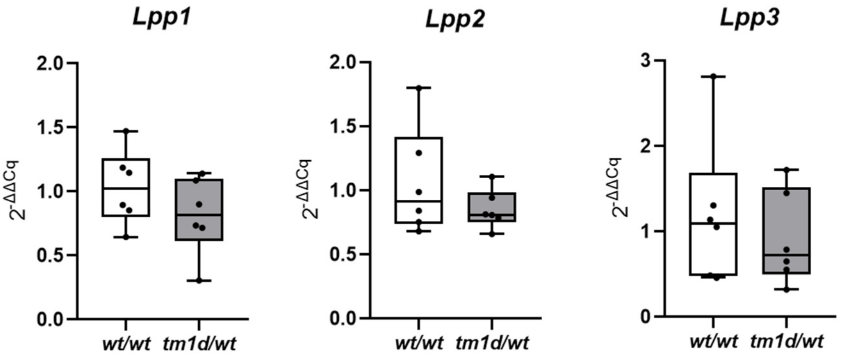

Figure S3: Lipid phosphate phosphatases (*Lpp*) profiling in kidneys of *Lpar1*$^{tm1d/WT}$ and control mice. Normalization was performed against *B2m*.

Table S1: *Lpar1*^{tm1a} in heterozygotic state does not lead to perinatal lethality. Observed and expected frequency of offspring genotypes from 5 independent matings of Tm1a+/- with C57BL/6J.

**Total matings**	**Total progeny**			
	**48 (100%)**			
	**tm1a$^{+/+}$**		**tm1a$^{+/-}$**	
**5**	**Born**	**Expected**	**Born**	**Expected**
	24	24	24	24
	50%	50%	50%	50%
**1♂ C57BL/6J x 1♀ tm1a$^{+/-}$**	**6**			
	4	3	2	3
	66.7%	50%	33.3%	50%
**1♂ tm1a$^{+/-}$ x 1♀ C57BL/6J**	**14**			
	6	7	8	7
	42.8%	50%	57.1%	50%
**1♂ tm1a$^{+/-}$ x 2♀ C57BL/6J**	**10**			
	4	5	6	5
	40%	50%	60%	50%
**1♂ tm1a$^{+/-}$ x 2♀ C57BL/6J**	**5**			
	3	2-3	2	2-3
	60%	50%	40%	50%
**1♂ tm1a$^{+/-}$ x 2♀ C57BL/6J**	**13**			
	7	6-7	6	6-7
	53.8%	50%	46.2%	50%

Table S2: *Lpar1^{tm1a}* in homozygotic state may lead to perinatal lethality. Observed and expected frequency of offspring genotypes from 3 independent matings of $Tm1a^{+/-}$ with $Tm1a^{+/-}$.

**Total matings**	**Total progeny**					
**3**	**12 (100%)**					
	**tm1a+/+**		**tm1a+/-**		**tm1a-/-**	
	**Born**	**Expected**	**Born**	**Expected**	**Born**	**Expected**
	4	2-3	7	5-6	1	2-3
	33.3%	25%	58.3%	50%	8.3%	25%
**1♂ tm1a/wt x 2♀ tm1a/wt**	**7**					
	1	1-2	5	3-4	1	1-2
	14.3%	25%	71.4%	50%	14.3%	25%
**1♂ tm1a/wt x 2♀ tm1a/wt**	**3**					
	3	1	0	1-2	0	1
	100%	25%	0%	50%	0%	25%
**1♂ tm1a/wt x 2♀ tm1a/wt**	**2**					
	0	0-1	2	1	0	0-1
	0%	25%	100%	50%	0%	25%

Table S3: *Lpar1^{tm1b}* in heterozygotic state may lead to perinatal lethality. Observed and expected frequency of offspring genotypes from 9 independent matings of $Tm1b^{+/-}$ with C57BL/6J.

**Total matings**	**Total progeny**			
**9**	**53 (100%)**			
	**$tm1b^{+/+}$**		**$tm1b^{+/-}$**	
	**Born**	**Expected**	**Born**	**Expected**
	39	26-27	14	26-27
	73.6%	50%	26.4%	50%
**1♂ C57BL/6J x 1♀ $tm1b^{+/-}$**	**5**			
	5	2-3	0	2-3
	100%	50%	0%	50%
**1♂ C57BL/6J x 2♀ $tm1b^{+/-}$**	**8**			
	7	4	1	4
	87.5%	50%	12.5%	50%
**1♂ C57BL/6J x 2♀ $tm1b^{+/-}$**	**7**			
	5	3-4	2	3-4
	71.4%	50%	28.6%	50%
**1♂ C57BL/6J x 2♀ $tm1b^{+/-}$**	**6**			
	5	3	1	3
	83.3%	50%	16.7%	50%
**1♂ C57BL/6J x 2♀ $tm1b^{+/-}$**	**9**			
	6	4-5	3	4-5
	66.7%	50%	33.3%	50%
**1♂ C57BL/6J x 1♀ $tm1b^{+/-}Cre^{-/-}$**	**2**			
	2	1	0	1
	100%	50%	0%	50%
**1♂ C57BL/6J x 1♀ $tm1b^{+/-}Cre^{-/-}$**	**8**			
	4	4	4	4
	50%	50%	50%	50%
**1♂ C57BL/6J x 2♀ $tm1b^{+/-}$**	**6**			
	3	3	3	3
	50%	50%	50%	50%
**1♂ C57BL/6J x 2♀ $tm1b^{+/-}$**	**2**			
	2	1	0	1
	100%	50%	0%	50%

Table S4: *Lpar1*^{tm1c} heterozygotes viability: Observed and expected frequency of offspring genotypes from 10 independent matings of Tm1c$^{+/-}$ with C57BL/6J.

**Total matings**	**Total progeny**			
**10**	**102 (100%)**			
	**tm1c$^{+/+}$**		**tm1c$^{+/-}$**	
	**Born**	**Expected**	**Born**	**Expected**
	56	51	46	51
	54.9%	50%	45.1%	50%
**1♂ C57BL/6J x 1♀ tm1c$^{+/-}$**	**3**			
	3	1-2	0	1-2
	100%	50%	0%	50%
**1♂ C57BL/6J x 1♀ tm1c$^{+/-}$**	**6**			
	2	3	4	3
	33.3%	50%	66.7%	50%
**1♂ tm1c$^{+/-}$ x 3♀ C57BL/6J**	**7**			
	5	3-4	2	3-4
	71.4%	50%	28.6%	50%
**1♂ tm1c$^{+/-}$ x 2♀ C57BL/6J**	**12**			
	7	6	5	6
	58.3%	50%	41.6%	50%
**1♂ C57BL/6J x 1♀ tm1c$^{+/-}$**	**9**			
	4	4-5	5	4-5
	44.4%	50%	55.6%	50%
**1♂ C57BL/6J x 2♀ tm1c$^{+/-}$**	**13**			
	7	6-7	6	6-7
	53.8%	50%	46.2%	50%
**1♂ C57BL/6J x 1♀ tm1c$^{+/-}$**	**7**			
	3	3-4	4	3-4
	42.9%	50%	57.1%	50%
**1♂ C57BL/6J x 2♀ tm1c$^{+/-}$ Flp$^{-/-}$**	**17**			
	10	8-9	7	8-9
	58.8%	50%	41.2%	50%
**1♂ C57BL/6J x 2♀ tm1c$^{+/-}$ Flp$^{-/-}$**	**16**			
	8	8	8	8
	50%	50%	50%	50%
**1♂ tm1c$^{+/-}$ Flp$^{-/-}$ x 2♀ C57BL/6J**	**12**			
	7	6	5	6
	58.3%	50%	41.6%	50%
